# Identification of ANXA3 as a biomarker associated with pyroptosis in ischemic stroke

**DOI:** 10.1186/s40001-023-01564-y

**Published:** 2023-12-15

**Authors:** Linquan Liu, Yahong Cai, Changqing Deng

**Affiliations:** 1 Chronic Disease Management Department, The First Hospital of Hunan University of Chinese Medicine, Changsha, 410007 Hunan China; 2 The Key Laboratory of Hunan Province for Integrated Traditional Chinese and Western Medicine on Prevention and Treatment of Cardio-Cerebral Diseases, College of Integrated Traditional Chinese and Western Medicine, Hunan University of Chinese Medicine, Changsha, 410208 Hunan China

**Keywords:** Ischemic stroke (IS), Pyroptosis, Random forest learning, Annexin A3 (ANXA3), ADM

## Abstract

**Background:**

Pyroptosis plays an important role in the pathological process of ischemic stroke (IS). However, the exact mechanism of pyroptosis remains unclear. This paper aims to reveal the key molecular markers associated with pyroptosis in IS.

**Methods:**

We used random forest learning, gene set variation analysis, and Pearson correlation analysis to screen for biomarkers associated with pyroptosis in IS. Middle cerebral artery occlusion/reperfusion (MCAO/R) and oxygen and glucose deprivation/reoxygenation (OGD/R) models were constructed in vitro and in vivo. Cells were transfected with an Annexin A3 silencing (si-ANXA3) plasmid to observe the effects of ANXA3 on OGD/R + lipopolysaccharides (LPS)-induced pyroptosis. qRT‒PCR and western blotting were used to detect the expression of potential biomarkers and pyroptotic pathways.

**Results:**

Samples from a total of 170 IS patients and 109 healthy individuals were obtained from 5 gene expression omnibus databases. Thirty important genes were analyzed by random forest learning from the differentially expressed genes. Then, we investigated the relationship between the above genes and the pyroptosis score, obtaining three potential biomarkers (ANXA3, ANKRD22, ADM). ANXA3 and ADM were upregulated in the MCAO/R model, and the fold difference in ANXA3 expression was greater. Pyroptosis-related factors (NLRP3, NLRC4, AIM2, GSDMD-N, caspase-8, pro-caspase-1, cleaved caspase-1, IL-1β, and IL-18) were upregulated in the MCAO/R model. Silencing ANXA3 alleviated the expression of pyroptosis-related factors (NLRC4, AIM2, GSDMD-N, caspase-8, pro-caspase-1, cleaved caspase-1, and IL-18) induced by OGD/R + LPS or MCAO/R.

**Conclusion:**

This study identified ANXA3 as a possible pyroptosis-related gene marker in IS through bioinformatics and experiments. ANXA3 could inhibit pyroptosis through the NLRC4/AIM2 axis.

**Supplementary Information:**

The online version contains supplementary material available at 10.1186/s40001-023-01564-y.

## Introduction

Ischemic stroke (IS) accounts for 75–80% of all strokes and is the leading cause of disability and death worldwide [1, 2]. Approximately 45% of IS cases are caused by blood clots in small or large arteries. Cardioembolic and lacunar strokes account for 14–30% and 15–25% of IS cases, respectively [3]. The different stages of the cascade reaction have been extensively studied. However, traditional treatment methods, such as antithrombotic therapy, neuroprotective drugs, or surgical implementation, are substantially limited due to poor safety or therapeutic efficacy [4, 5]. Therefore, uncovering the underlying molecular mechanisms and exploring innovative therapeutic targets for IS have always been a top priority.

Pyroptosis, a type of programmed cell death, is accompanied by the release of a large number of inflammatory factors [6]. During IS, inflammasomes, such as abstract in melanoma 2 (AIM2), NLR-family CARD-containing protein 4 (NLRC4), and PYD domain-containing protein 3 (NLRP3), are activated in cells [7, 8]. This process further activates caspase-1. Caspase-1 activates the cleavage of the N-terminal sequence of Gasdermin-D (GSDMD), allowing it to bind to the membrane and generate membrane pores. Similarly, caspase-1 can simultaneously cleave pro-IL-1β and pro-IL-18 into biologically active forms, producing mature proinflammatory cytokines, which are released into the extracellular environment. Proinflammatory cytokines are toxic to neural cells, leading to pyroptosis [6]. Recent studies have shown that caspase-8 can also participate in pyroptosis by cleaving GSDMD [9, 10]. A combination of several antiplatelet drugs has been shown to attenuate inflammasome-mediated pyroptosis by inhibiting the NF-κB/NLRP3 pathway in IS [6, 11]. However, there are few studies of pyroptosis-related biomarkers in IS.

In this study, based on bioinformatics and experimental verification methods, the analysis of differentially expressed genes (DEGs) between healthy individuals and IS patients was used to reveal the mechanisms related to pyroptosis, identify new potential therapeutic targets, and investigate pyroptosis in IS. The foundation was laid for the development of conditioning treatment regimens.

## Materials and methods

### Processing of data and computational analysis

The Gene Expression Omnibus (GEO) database (http://www.ncbi.nlm.nih.gov/geo) was used to obtain gene expression profiles of IS, including GSE58294, GSE22255, GSE66724, GSE16561, and GSE37587. The details of the dataset are presented in Additional file 1: Table S1. Among them, GSE58294, GSE22255, and GSE66724 are all GPL570 platforms integrated into one dataset. GSE16561 and GSE37587 are GPL6883 platforms integrated into one dataset. The corresponding dataset is represented by the platform number. For multiple probes corresponding to a gene, the average expression value was taken as the gene expression value. ComBat was utilized to eliminate batch-to-batch variation in the "sva" R package. Distribution patterns of IS and control samples (before and after batch correction using ComBat) were observed by principal component analysis (PCA).

### Screening of pyroptosis-related DEGs

The two datasets were analyzed for differences using the limma package. The filter condition was |logFC|> log_2_(1.5) and p < 0.05. The filtered DEGs were used for random forest analysis to obtain the important common genes of the two datasets. The pyroptosis-associated gene set, including 44 genes, is shown in Additional file 2: Table S2. After gene set variation analysis (GSVA), pyroptosis scores were obtained. Correlation analysis was performed using the obtained important common genes and pyroptosis scores in the two datasets. The genes with R > 0.2 and p < 0.05 were included in the next analysis, and the intersection of the two data sets was taken as potential pyroptosis-related genes in IS.

### Animals

A total of 48 adult male Sprague–Dawley rats (250–280 g) were purchased from the Hunan Slack Jingda Experimental Animal Co., Ltd. All rats underwent a 12 h light–dark cycle in a room with constant humidity (40–70%) and temperature (22–26 °C). Rats were allowed to eat and drink freely before the experiment and fasted for 12 h before surgery. Animals were randomly divided into 2 groups, including the sham operation group (Sham, n = 8) and the middle cerebral artery occlusion/reperfusion (MCAO/R) model group (n = 8). The MCAO/R model was constructed as previously described [11]. Rats were anesthetized by intraperitoneal injection of sodium pentobarbital (35 mg/kg). Rats were fixed in the supine position. Nylon (0.26 mm in diameter, 40 mm in length) was used to suture the internal carotid artery. The indwelling sutures were maintained for 2 h, and the filaments were removed to allow reperfusion for 24 h. During MCAO/R, the body temperature of the rats was maintained at 37 ± 0.5 °C. In the sham group, the same surgical induction was performed, but the artery was not ligated.

As previously described [12, 13], 3 days before the MCAO/R intervention, rat brains were targeted for slow injection of 10 µl of 4 × 10^9^ TU/ml sh-NC and sh-ANXA3 lentiviral suspensions further to explore the effects of ANXA3 on the animal model. The injection process was carried out at 0.2 μl/min. Following the injection, the rats were subjected to MCAO/R treatment. The rats in the model and sham-operated groups were treated as described above.

All experiments were performed in accordance with institutional guidelines. Rats were sacrificed by intraperitoneal injection of 150 mg/kg sodium pentobarbital. Brain tissue and peripheral blood were obtained for subsequent experimental detection. This study was approved by the Animal Ethical and Welfare Committee of Hunan University of Traditional Chinese Medicine (LL2021091501).

### Neurological defect scoring

The neurological function of rats was scored according to Bederson's method [14]. The rat tail was suspended. Forelimb flexion was observed. A score of 0 points indicated that the activity of the rat was normal; 1 point indicated that when the rat was lifted vertically, the left forelimb could not be fully extended; 2 points indicated that the lateral thrust resistance of the rat was reduced; 3 points indicated unilateral rotation when the rat walked freely; and 4 points indicated that the rat was unable to walk due to flaccid paralysis. A higher score indicates worse neurological function.

### Infarct size measurement

Brain tissue was cut into 5 consecutive coronal brain sections. Afterward, brain slices were incubated with 1% 2,3,5 triphenyltetrazolium chloride (TTC, Sangon Biotech Co., Ltd.) for 15 min at 37 °C in the dark [15]. With reference to previous work [16, 17], the formula for calculating the percentage of infarcted area was calculated as follows: infarct rate (%) = (volume of the unlesioned hemisphere—volume of the lesioned hemisphere that is not infarcted)/volume of the unlesioned hemisphere × 100%.

### Immunofluorescence (IF) staining

Tissue sections were grilled as previously described [18] and then deparaffinized to water. After heat antigen repair and routine pre-treatments, sections were incubated overnight at 4 °C with primary antibody (caspase1, 22915–1-AP, 1:100, Proteintech, USA). Then, the sections were incubated with a secondary antibody (Goat Anti-Rabbit IgG(H + L), SA00013-2, Proteintech, USA) for 90 min at 37 °C. After nucleation and sealing, the sections were placed under a fluorescence microscope (XD-202, Ningbo Jiangnan Instrument Factory, China) for observation. Image J (ImageJ, National Institutes of Health, USA) was used to analyze the fluorescence intensity.

### Cell treatment

A rat pheochromocytoma cell line (PC12, CL-0412, Pricella, China) was cultured in Dulbecco's modified Eagle's medium (D5796, DMEM, Gibco, USA) containing 10% fetal bovine serum (FBS, 10,099,141, Gibco, USA). Cells were cultured at 37 °C in a 5% CO_2_ environment.

Cells were first divided into control, oxygen–glucose deprivation/reoxygenation (OGD/R), and OGD/R + lipopolysaccharide (LPS) groups. The cells in the OGD/R group were placed in sugar-free DMEM under hypoxia (95% N2, 5% CO_2_) for 2 h and then reoxygenated for 2 h. The cells in the OGD/R + LPS group were placed in sugar-free DMEM containing LPS (1 μg/mL, 82857-67-8, Sigma, USA) under hypoxia (95% N2, 5% CO_2_) for 2 h and then reoxygenated for 2 h [11].

Cells were divided into the control, OGD/R + LPS, OGD/R + LPS + silencing of negative control (si-NC, Invitrogen, USA), and OGD/R + LPS + silencing of Annexin A3 (si-ANXA3) groups. Cells in the OGD/R + LPS group were treated as above. Cells in the OGD/R + LPS + si-NC and OGD/R + LPS + si-ANXA3 groups were treated as follows. After cells were transfected with si-NC and si-ANXA3 with Lipofectamine 2000 (2028090, Invitrogen, USA), they were treated with OGD/R and LPS.

### Cell counting kit-8 (CCK-8)

The cell viability was assessed using CCK-8 solution (10%) following the manufacturer’s instructions (AWC0114a, Abiowell, China). After incubating the cells at 37 °C with 5% CO_2_ for 4 h, the absorbance (OD) value at 450 nm was measured using an enzyme marker (MB-530, HEALES, China).

### Quantitative real-time PCR (qRT‒PCR)

The above total RNA from peripheral blood, tissue, or cells was extracted and quantitatively analyzed. One microgram of RNA was used to synthesize cDNA. An UltraSYBR Mixture kit (CW2601, CWBIO, China) was utilized as a template for PCR. The primers are as follows. ANXA3, forward primer: TCAGCTCTCTGAGCCTTAGGT; reverse primer: CTCGTGGGGTGACCATTTCG; ANKRD22, forward primer: CAAAGCAGAATGAGGCTCTCG; reverse primer: GTAGCCGTAGCAGTCGGTAG; ADM, forward primer: TTGGACTTTGCGGGTTTTGC, reverse primer: GCTCCGATACCCTGCTGAAA; GAPDH, forward primer: ACAGCAACAGGGTGGTGGAC; reverse primer: TTTGAGGGTGCAGCGAACTT. The expression level of the target gene was analyzed by the 2^−ΔΔCT^ method. Amplified PCR products were quantified and normalized to GAPDH as a control.

### Western blotting

The total protein from the tissues or cells described above was extracted and quantitatively analyzed. Protein homogenates were run on SDS‒PAGE gels and transferred to PVDF membranes. After the membrane was blocked with 5% milk, it was incubated overnight in the primary antibody (Additional file 3: Table S3). β-actin was conducted as an internal reference. The secondary antibody was incubated for 90 min at 37 °C. Membranes were subjected to enhanced chemiluminescence for signal intensity quantification. A chemiluminescence imaging system (ChemiScope 6100, China) was adopted for analysis.

### Functional enrichment analysis of ANXA3

The GSVA package was used for Gene Ontology (GO) and Kyoto Encyclopedia of Genes and Genomes (KEGG) analyses. Correlations between ANXA3 expression and pathways were analyzed. Important pathways among them were screened. Correlations between ANXA3 expression and all genes were analyzed. A gene set of 22 immune cells was utilized for immune cell analysis.

### Statistical analysis

Quantitative analysis was performed using Prism 8.0. A Pearson's coefficient correlation test was used to measure correlations. The level of significance was set at *p < 0.05. The error bar represents mean ± standard deviation.

## Results

### Screening of DEGs

**Fig. 1:**
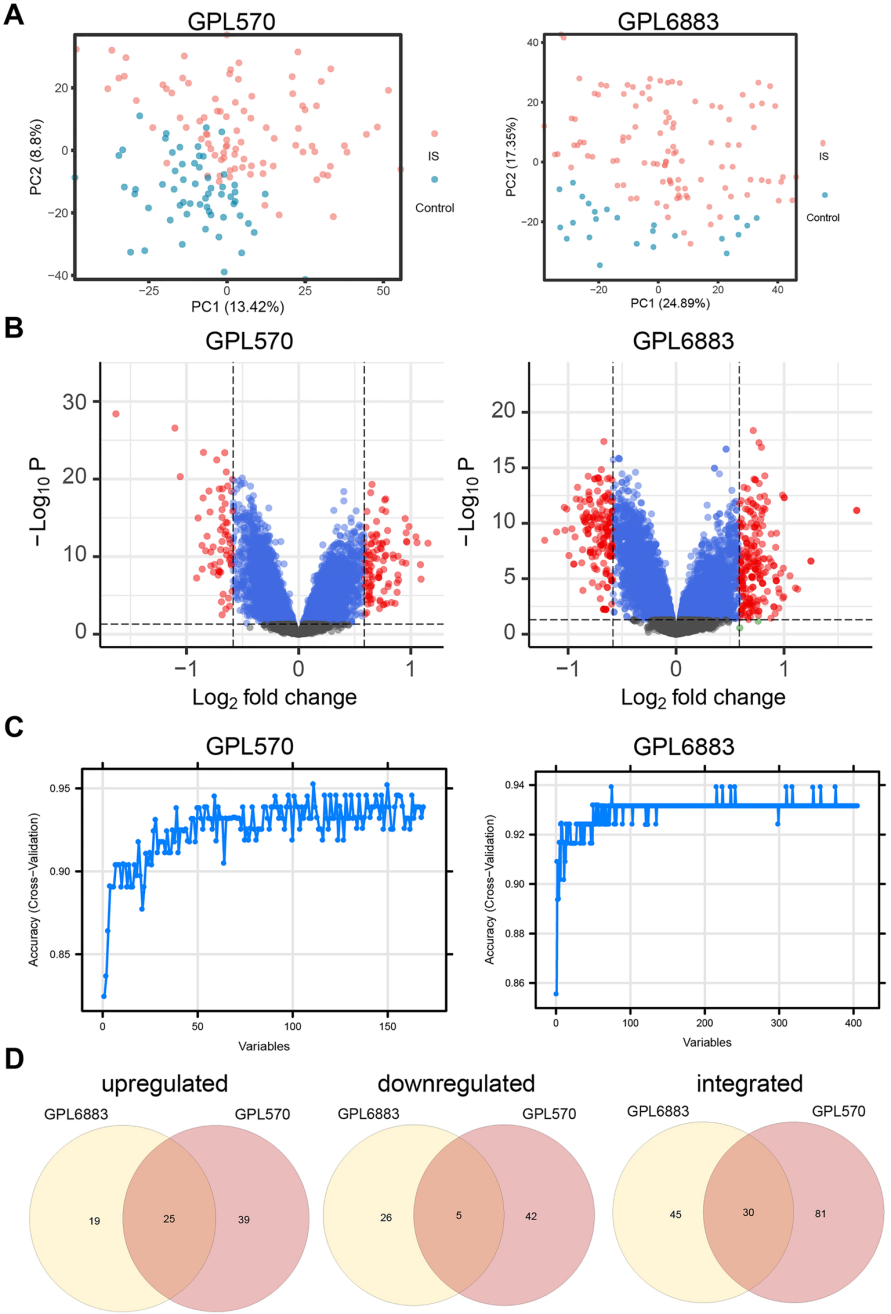
Screening of DEGs. **A** Scatter plot of PCA; **B** Volcano plot; **C** Random forest variables plot; **D** Venn diagram of important DEGs in the two datasets

The flow diagram of the study is shown in Additional file 4: Fig. S1. The scatter plots of PCA for the two datasets showed two different distribution patterns for the control and IS groups. The IS group was mainly distributed on the upper side of the figure, while the control group was mainly distributed on the lower side of the figure (Fig. 1A). The two datasets GPL570 and GPL6883 (samples from a total of 170 IS patients and 109 healthy individuals) were subjected to differential analysis using the limma package. The filter range was |logFC|> log2(1.5) and p < 0.05. Volcano plot results revealed DEGs (Fig. 1B). Subsequently, random forest analysis was used to screen important DEGs (Fig. 1C). Then, these genes were intersected to obtain 25 upregulated and 5 downregulated important DEGs (Fig. 1D). Next, these 30 genes were used for subsequent analysis.

### Screening of DEGs associated with pyroptosis

**Fig. 2:**
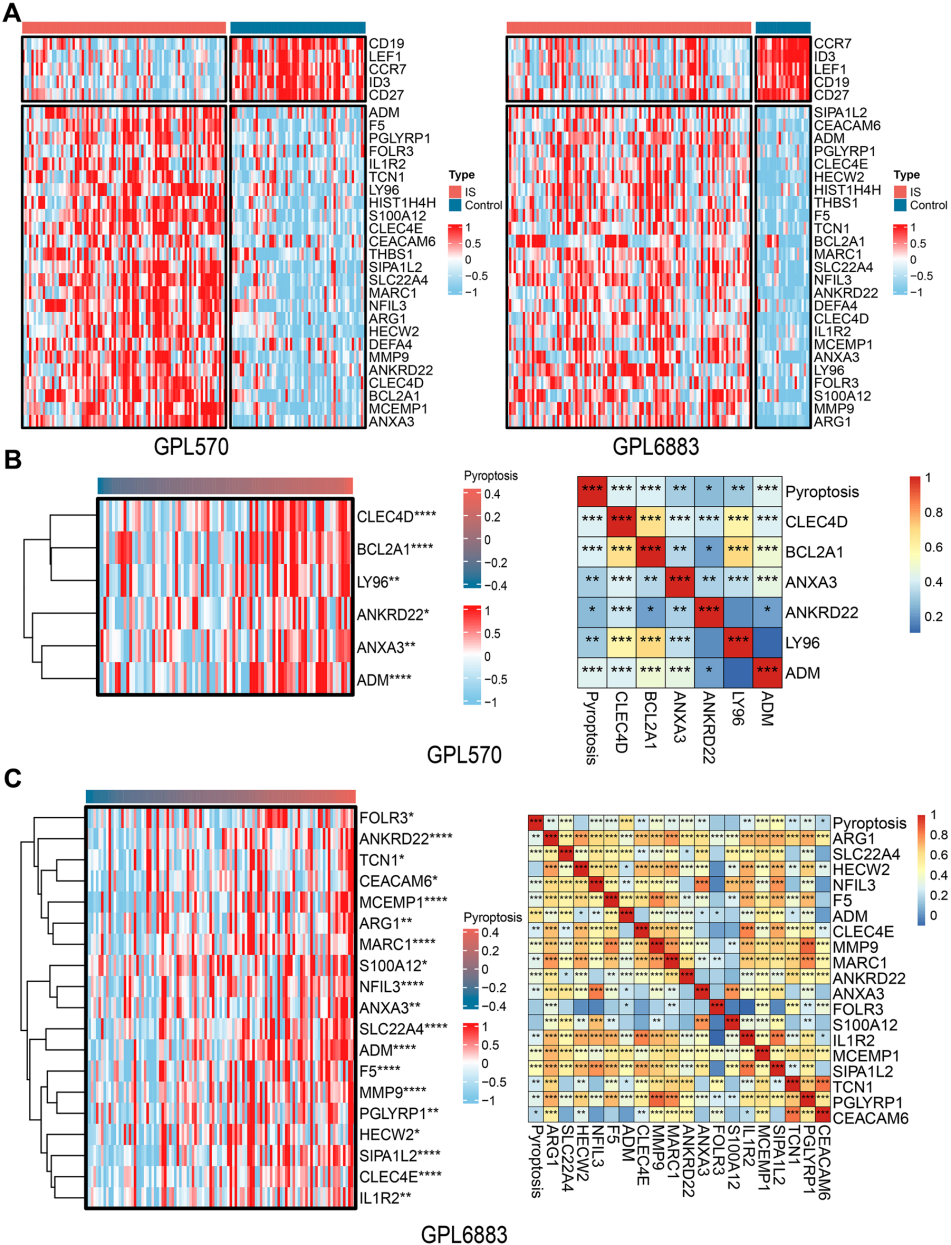
Screening of DEGs associated with pyroptosis. **A** Heatmap of 30 DEGs; **B**, **C** Heatmap of pyroptosis-related DEGs and correlation analysis in the GPL570 and GPL6883 datasets

**Fig. 3:**
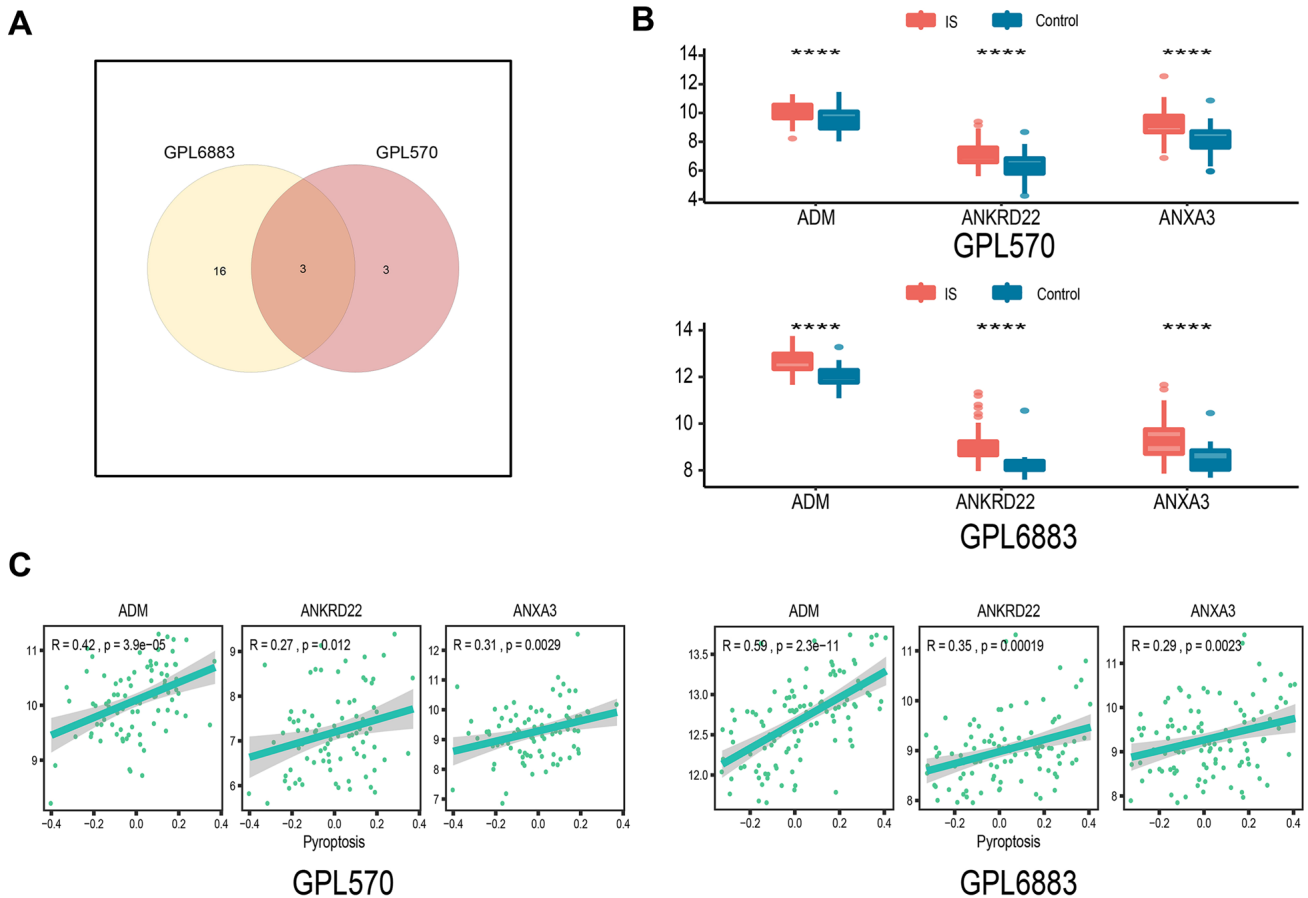
Screening of common DEGs associated with pyroptosis. **A** The intersection of screened important and pyroptosis-related genes in the two datasets; **B** The expression difference of pyroptosis-related genes in the IS and control groups; **C** Correlation analysis of pyroptosis-related genes and pyroptosis scores

The results of the above 30 DEGs in the control and IS groups are shown in the heatmap (Fig. 2A). The pyroptosis-related gene set was subjected to GSVA to obtain a pyroptosis score. In the GPL570 and GPL6883 datasets, the expression correlation between the pyroptosis-related gene set and the pyroptosis score is shown in Additional file 5: Fig. S2A and B. Correlation analysis was performed with the obtained important DEGs and pyroptosis scores in the two datasets, and 6 and 19 important genes related to pyroptosis were obtained. As shown in Fig. 2B, C, the expression of all the abovementioned important pyroptosis-related genes, such as ADM, CLEC4D, SIPA1L2, SLC22A4, and MCEMP1, significantly changed with the change in pyroptosis scores. The correlation analysis showed that the expression of all the above genes, such as CLEC4D (R = 0.38, p = 0.0002), SIPA1L2 (R = 0.52, p = 1.23e−08), SLC22A4 (R = 0.41, p = 1.74e−05), and MCEMP1 (R = 0.39, p = 3.94e−05), was positively correlated with the pyroptosis score. Genes with R > 0.2 and p < 0.05 were included in the next analysis. The intersection of the two datasets included three pyroptosis-related DEGs, ANXA3, ANKRD22, and ADM (Fig. 3A). ANXA3, ANKRD22, and ADM were significantly higher in the IS group than in the control group (Fig. 3B, p < 0.0001). In the GPL570 dataset, pyroptosis was positively correlated with the ANXA3 (R = 0.42, p = 3.9e−05), ANKRD22 (R = 0.27, p = 0.012), and ADM (R = 0.31, p = 0.0029) genes. Similarly, in the GPL6883 dataset, pyroptosis was positively correlated with the ANXA3 (R = 0.59, p = 2.3e−11), ANKRD22 (R = 0.35, p = 0.00019), and ADM (R = 0.29, p = 0.0023) genes (Fig. 3C). The above results suggest that the DEGs (ANXA3, ANKRD22, and ADM) in IS may be related to pyroptosis.

### ANXA3 is highly expressed in the MCAO/R rat model

**Fig. 4:**
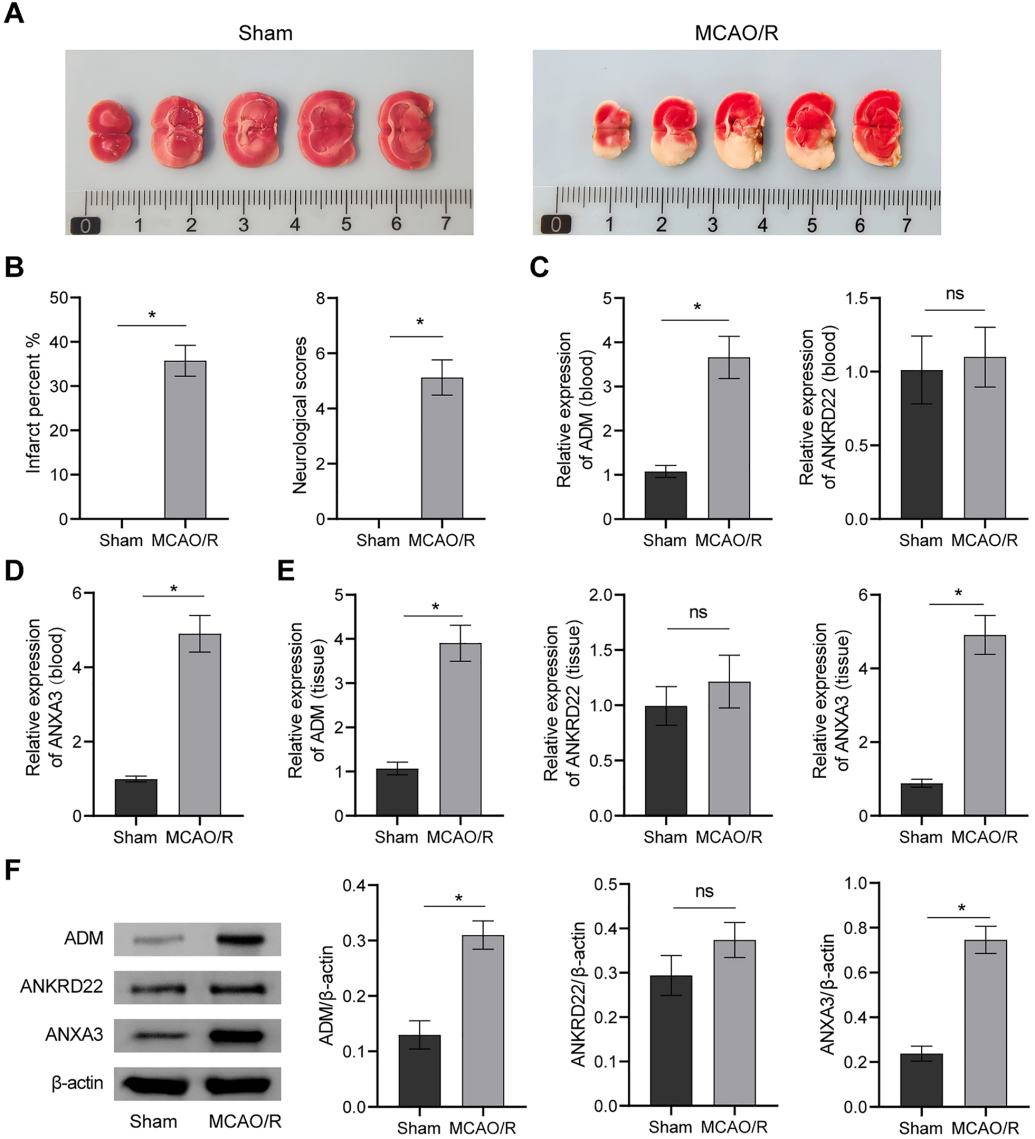
Expression validation of the common DEGs. **A** Representative pictures of the cerebral infarction area; **B** Quantitative statistics of the infarct percent and neurological function score; **C**, **D** Histogram of gene expression of ADM, ANKRD22, and ANXA3 in peripheral blood; **E**, **F** Histogram of ADM, ANKRD22, and ANXA3 gene and protein expression in brain tissue. *p < 0.05; ns means no significance

To further verify whether the above genes were differentially expressed in IS, we constructed a rat MCAO/R model. Compared with the rats in the sham group, the rats in the MCAO/R group developed large-area cerebral infarction. Neurological scores of the rats in the MCAO/R group were significantly higher than those of the sham group (Fig. 4A, B). After modeling, the expression levels of ADM and ANXA3 in peripheral blood and brain tissue were overtly increased (p < 0.05), and the fold difference in ANXA3 expression was larger. However, the expression of ANKRD22 was not significantly different (Fig. 4C–F), suggesting that the expression of ANXA3 and ADM might be associated with the development of IS.

**Fig. 5:**
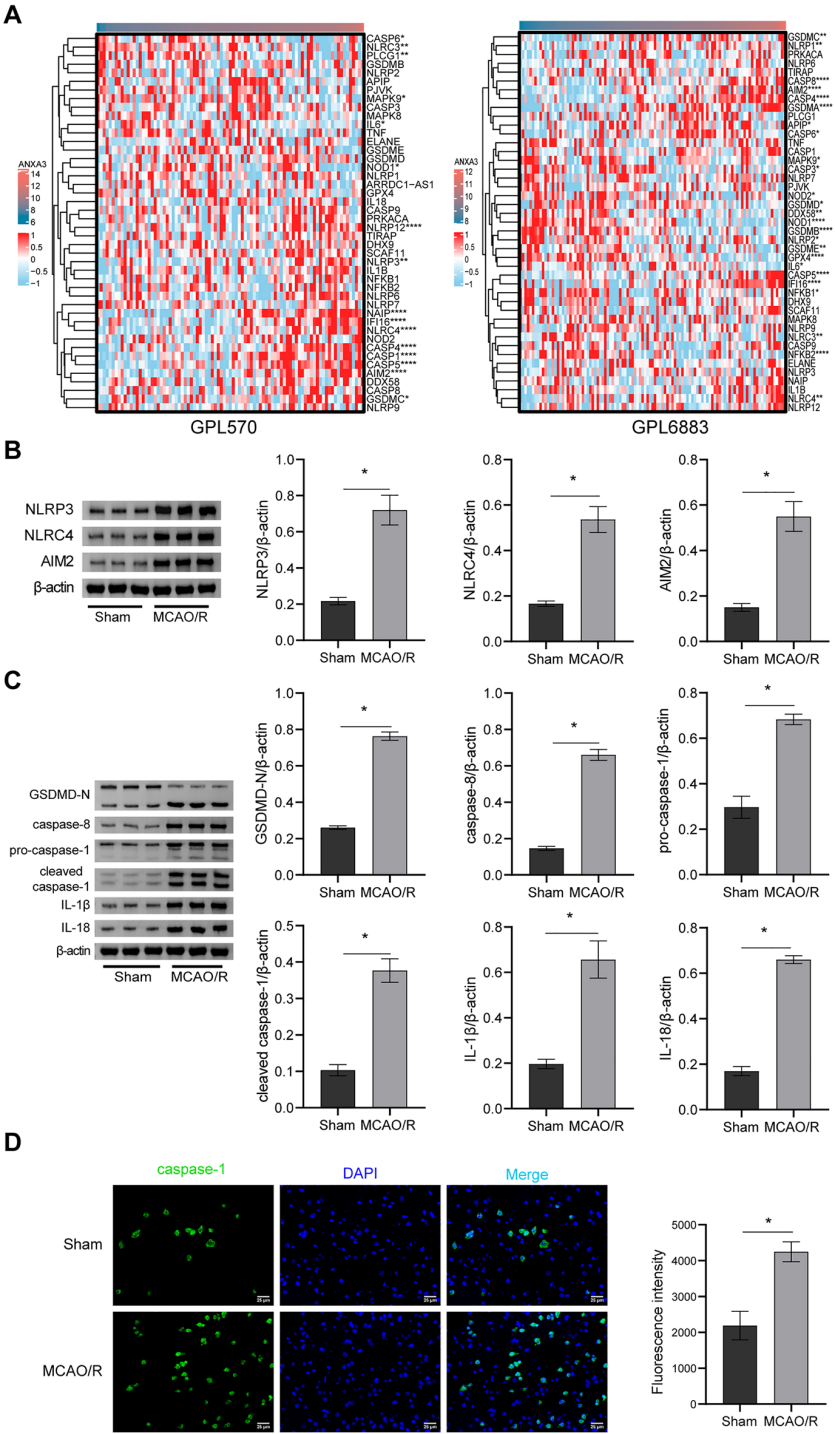
Pyroptosis occurs in the MCAO/R rat model. **A** Heatmap of the correlation between ANXA3 and pyroptosis-related gene set genes; **B**, **C** NLRP3, NLRC4, AIM2, GSDMD-N, caspase-8, pro-caspase-1, cleaved caspase-1, and IL-1β protein expression and quantitative statistics; **D** Representative images of caspase-1 staining and quantitative statistics. *p < 0.05

To further explore the association of ANXA3 with pyroptosis-related pathways, we analyzed the association of ANXA3 with the genes in the pyroptosis-related gene set. In both datasets, the expression of ANXA3 was significantly changed with changes in pyroptosis scores, such as those in NLRP12, NAIP, IFI16, NLRC4, CASP8, and AIM2 (Fig. 5A). The protein expression levels of NLRP3, NLRC4, AIM2, GSDMD-N, caspase-8, pro-caspase-1, cleaved caspase-1, caspase-1, IL-1β, and IL-18 were significantly increased in the rats in the MCAO/R group (Fig. 5B–D, p < 0.05), suggesting that pyroptosis occurred in the brain tissue of the model group.

### Inhibition of ANXA3 affects OGD/R + LPS-induced pyroptosis

**Fig.6:**
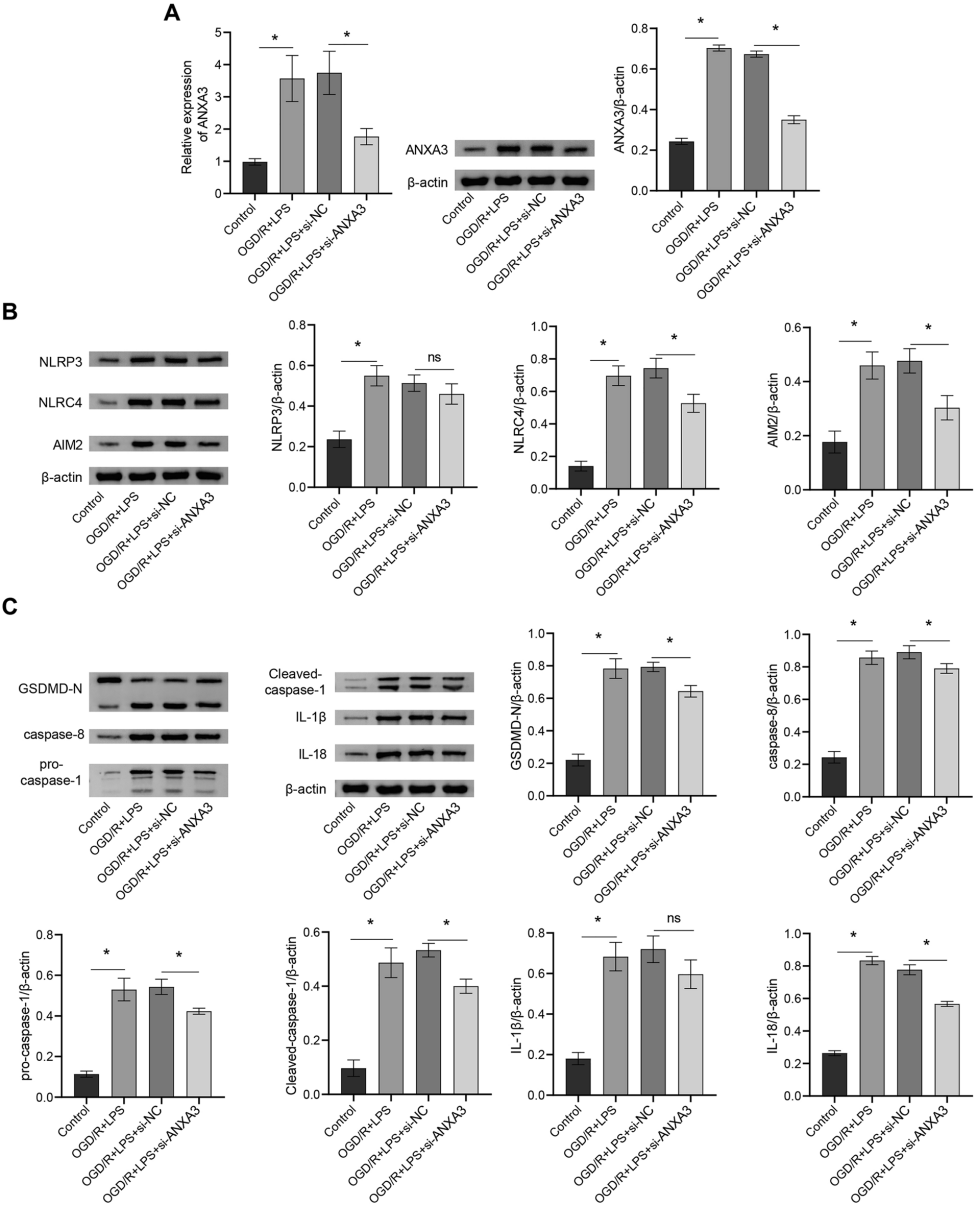
Inhibition of ANXA3 affects OGD/R + LPS-induced pyroptosis. **A** Gene and protein expression of ANXA3; **B**, **C** NLRP3, NLRC4, AIM2, GSDMD-N, caspase-8, pro-caspase-1, cleaved caspase-1, IL-1β, and IL-18 protein expression and quantitative statistics. *p < 0.05; ns means no significance

Next, we conducted in vitro studies. Cell activity was significantly inhibited in both the OGD/R + LPS and OGD/R groups compared to the control group, with the OGD/R + LPS group showing a greater level of inhibition (Additional file 6: Fig. S3A). Furthermore, both OGD/R and OGD/R + LPS induced the expression of GSDMD-N and IL-1β, with the highest expression observed in the OGD/R + LPS group (Additional file 6: Fig. S3B). Consequently, the OGD/R + LPS group was chosen for constructing in vitro cell models in the subsequent experiments. To further explore whether ANXA3 regulates pyroptosis-related pathways, we interfered with ANXA3 in cells. Si-ANXA3 inhibited the OGD/R combined with an LPS-induced increase in ANXA3 expression (Fig. 6A, p < 0.05). Si-ANXA3 significantly inhibited the OGD/R combined with LPS-induced increases in the expression of NLRC4, AIM2, GSDMD-N, caspase-8, pro-caspase-1, cleaved caspase-1, and IL-18 (Fig. 6B, C,  p < 0.05), suggesting that si-ANXA3 could inhibit the pyroptosis induced by OGD/R + LPS.

### Inhibition of ANXA3 affects MCAO/R-induced pyroptosis

**Fig. 7:**
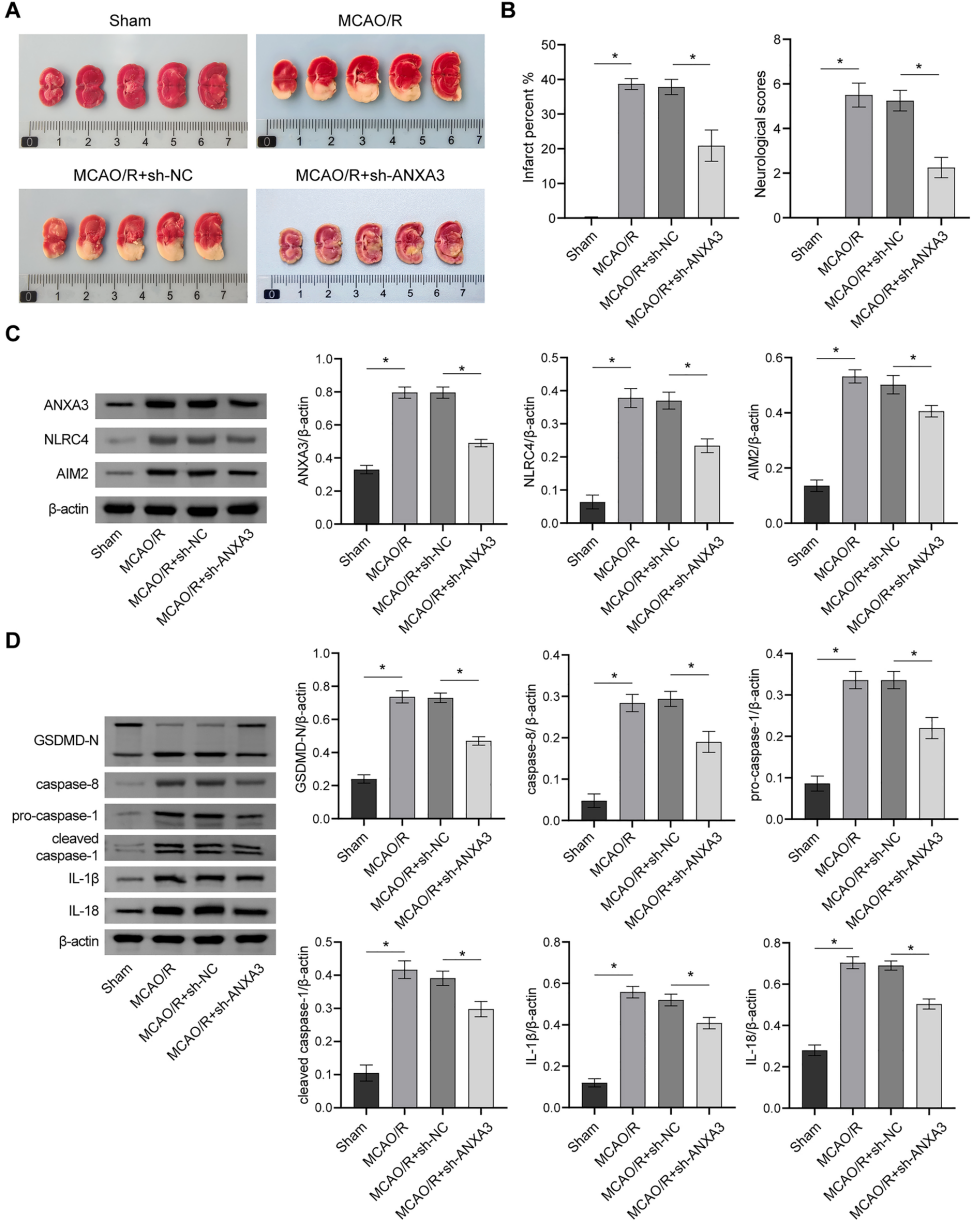
Inhibition of ANXA3 affects MCAO/R-induced pyroptosis. **A** Representative pictures of the cerebral infarction area; **B** Quantitative statistics of the cerebral infarct percent and neurological scores; **C**, **D** NLRP3, NLRC4, AIM2, GSDMD-N, caspase-8, pro-caspase-1, cleaved caspase-1, and IL-1β protein expression and quantitative statistics. *p < 0.05

To further validate the impact of ANXA3 inhibition on MCAO/R-induced animal models, we conducted in vivo experiments to suppress ANXA3 expression. The MCAO/R + sh-ANXA3 group showed lower infarct rate and neurological scores than the MCAO/R + sh-NC group (Fig. 7A and B). Additionally, the protein expression levels of NLRP3, NLRC4, AIM2, GSDMD-N, caspase-8, pro-caspase-1, cleaved caspase-1, and IL-1β were lower in the MCAO/R + sh-ANXA3 group as compared to the MCAO/R + sh-NC group (Fig. 7C and D). These findings indicated that ANXA3 inhibition could effectively suppress MCAO/R-induced pyroptosis.

### Functional enrichment analysis of ANXA3

**Fig. 8:**
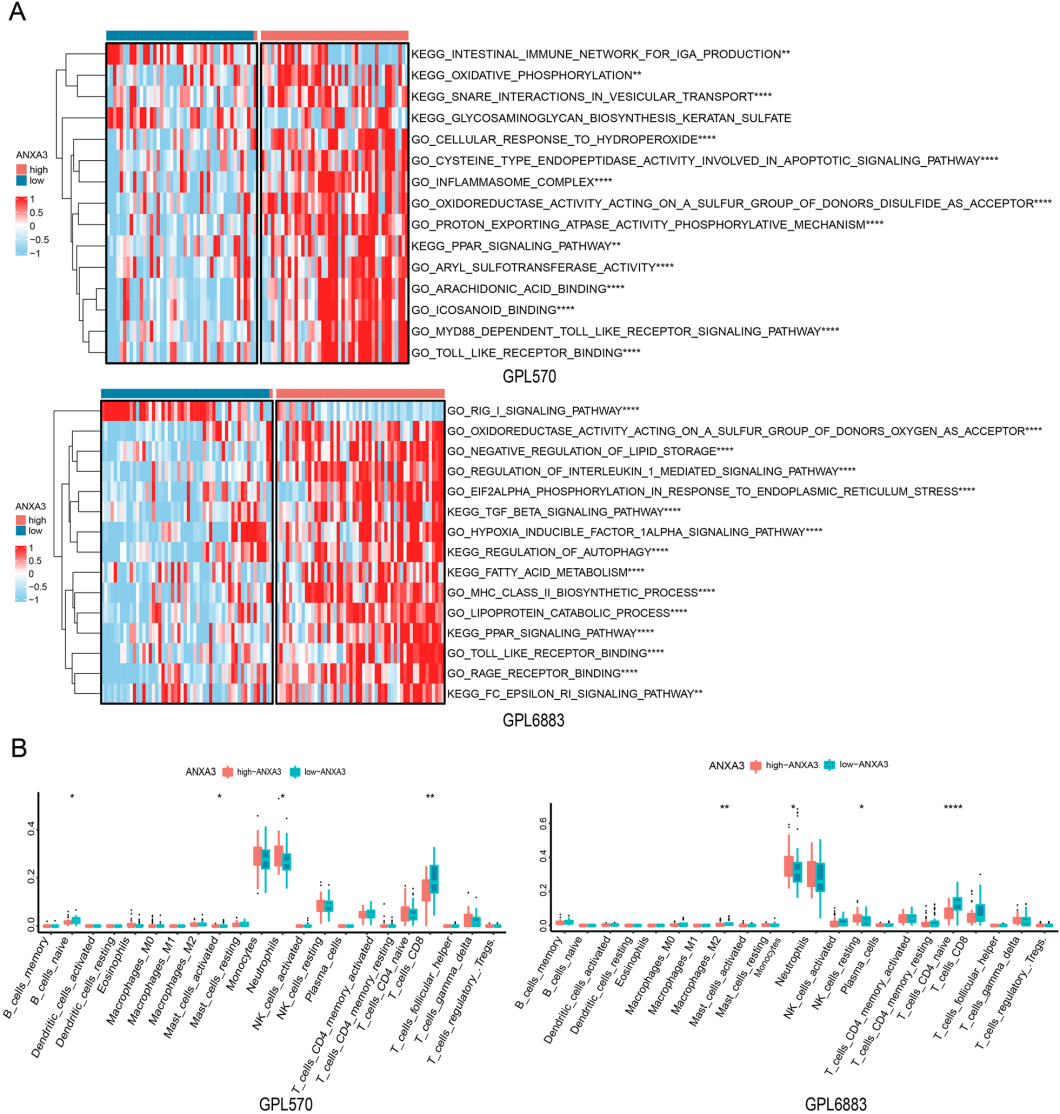
Functional enrichment analysis of ANXA3. **A** GO and KEGG analyses; **B** immune infiltration analysis of the low- and high-ANXA3 groups

We further analyzed the functional enrichment of the ANXA3 gene. ANXA3 was mainly enriched in Toll-like receptor binding, inflammasome complex, MYD88-dependent Toll-like receptor signaling pathway, oxidoreductase activity acting on a sulfur group of donors’ oxygen as acceptor, regulation of interleukin 1-mediated signaling pathway, hypoxia inducible factor 1alpha signaling pathway, and TGF beta signaling pathway (Fig. 8A). In addition, in the functional enrichment analysis of immune cells, compared with the low ANXA3 group, the high ANXA3 group showed lower levels of CD8 T cells (p < 0.01), M2 macrophages (p < 0.01), and naive CD4 T cells (Fig. 8B, p < 0.0001). The above results suggest that ANXA3 may be associated with inflammatory and immune regulatory pathways.

## Discussion

Targeting pyroptosis to modulate IS damage is a potential therapeutic strategy [19]. Based on random forest analysis, GSVA, and Pearson correlation analysis, this study identified three DEGs that might be associated with pyroptosis in IS: ADM, ANKRD22, and ANXA3. ADM and ANXA3 were significantly overexpressed in the MCAO/R model, and the fold difference in ANXA3 expression was greater. Pyroptosis-related proteins were highly expressed in the MCAO/R model. Silencing ANXA3 could reverse the expression of NLRC4 and AIM2 pyroptotic inflammasomes, suggesting that inhibition of ANXA3 might inhibit pyroptosis through the NLRC4/AIM2 axis in PC12 cells.

Bioinformatics is an efficient biomarker screening method [20]. Li et al. screened immune-related genes in IS based on bioinformatics methods [21]. Machine learning techniques are increasingly used in the medical field due to their high accuracy [22]. Random forest is a type of machine learning and is a common way to screen important marker genes [23]. Chen et al. identified DEGs associated with ferroptosis in IS through bioinformatics analysis of the GSE16561 and GSE22255 datasets [24]. Martha et al. used a random forest algorithm to predict the prognostic biomarkers of stroke in patients with IS [25]. Through weighted gene coexpression network analysis and machine learning, the ADM, ANXA3, SLC22A4, and VIM genes were predicted to be serum markers associated with immune cell infiltration for the diagnosis of IS [26]. In this study, based on random forest, GSVA, and Pearson correlation analyses, three genes possibly related to IS pyroptosis were screened: ANXA3, ANKRD22, and ADM.

ANKRD22, a nuclear-encoded mitochondrial protein, can be involved in gastric mucosal injury and various cancer processes, such as colorectal cancer and prostate cancer [27–29]. In a study of Parkinson's disease, ANKRD22 obtained through bioinformatics screening was shown to regulate neuronal development by regulating cell viability and IL-6 expression [30]. ADM, which acts as a vascular inhibitor, is upregulated in major depressive disorder, suggesting that ADM may be associated with neuroinflammation-related diseases [31]. ANXA3 has been studied in various neuroinflammatory diseases, such as schizophrenia, traumatic brain injury, and cerebral small vessel disease [32–34]. Current studies suggest that ANKRD22, ADM, and ANXA3 may serve as markers associated with pyroptosis in IS. ANXA3 is an upregulated protein in the brains of IS patients and rats with MCAO/R [35, 36]. ADM and ANXA3 may be potential immune-related markers for the diagnosis of IS [26]. Similar to the above studies, our study found that ADM and ANXA3 were upregulated in the brain tissue and peripheral blood of the rats in the MCAO/R group.

Studies on the regulation of pyroptosis by the NLRP3 inflammasome in IS are more frequently reported than those on the NLRC4/AIM2 inflammasome. For example, TMEM59, a type I transmembrane protein, is protective against IS by inhibiting pyroptosis and inflammatory responses via NLRP3/ASC/cleaved caspase-1 [37]. Hypoxia-inducible factor-1α (HIF-1α) may modulate the inflammatory response through the NLRP3 inflammasome complex, thereby affecting pyroptosis after stroke [38]. Medioresinol, a PGC-1α activator, reduces cerebral infarct volume and blood‒brain barrier permeability through the PPARα/GOT1 axis, inhibits endothelial cell pyroptosis, and promotes long-term neurobehavioral recovery [39]. Recent studies have shown that the NLRC4 inflammasome complex mediates the inflammatory response and pyroptosis of microglia in vitro and in vivo under ischemic conditions [8]. Long noncoding RNA MEG3 promotes cerebral ischemia‒reperfusion injury by targeting the miR-485/AIM2 axis to increase pyroptosis [40]. Similarly, in this study, we found that inflammasome (NLRP3, NLRC4, and AIM2 proteins) and pyroptosis-related downstream proteins (GSDMD-N, caspase-8, pro-caspase-1, cleaved caspase-1, IL-1β, and IL-18) were upregulated in the MCAO/R model. ANXA3 pathway inhibition can protect against brain MCAO/R injury [41]. More importantly, we found that inhibition of ANXA3 might inhibit pyroptosis by inhibiting the NLRC4/AIM2 pathway but not the NLRP3 pathway.

Toll-like receptor binding, inflammasome complex, MYD88-dependent Toll-like receptor signaling pathway, regulation of interleukin 1-mediated signaling pathway, and TGF beta signaling pathway are all pathways closely related to neuroinflammatory and pyroptotic responses. For example, the Toll-like receptor binding pathway can be involved in neutrophil infiltration and polarization in IS and is also associated with cytokine secretion and phagocytic activity of microglia in the central nervous system [42, 43]. Blockade of the TLR2/TLR4-MyD88 pathway inhibits neuroinflammation in mice with MCAO/R [44]. Through a cascade of signaling pathways in TLRs, NLRP3 is activated to induce the production of IL-1β and IL-18, leading to pyroptosis [45]. Activation of the TGF-β/NLRP3/caspase-1 signaling pathway stimulates pyroptosis, which ultimately exacerbates sepsis-induced acute kidney injury [46]. Therefore, ANXA3 is enriched in the pathways mentioned earlier, suggesting that ANXA3 may be related to inflammation and immune regulation pathways in IS. Further correlation analysis of immune cell infiltration showed that the low ANXA3 group was enriched in CD8 T cells, M2 macrophages, and naive CD4 T cells. The above results suggest that ANXA3 may be a key gene related to inflammation, pyroptosis, and immunity.

However, we did not further explore the detailed mechanism by which inhibition of ANXA3 suppresses MCAO/R-induced pyroptosis, which is a limitation of our study. We plan to do further mechanistic exploration using gene chips and other in vivo and in vitro experiments in future studies.

## Conclusion

This study identified ANXA3 as a possible pyroptosis-related gene marker in IS through bioinformatics and experiments. ANXA3 can inhibit pyroptosis through the NLRC4/AIM2 axis in PC12 cells.

### Supplementary Information

**Additional file 1: Table S1.** Basic information of gene expression profiling.

**Additional file 2: Table S2.** Pyroptosis-related gene sets.

**Additional file 3: Table S3.** Antibody information.

**Additional file 4: Fig. S1.** Flow diagram of the study.

**Additional file 5: Fig. S2.** Expression correlation analysis of the pyroptosis-related gene set in the GPL570 and GPL6883 datasets. (A) GPL570; (B) GPL6883.

**Additional file 6: Fig. S3.** OGD/R+LPS promotes cellular pyroptosis. (A) CCK-8; (B) GSDMD-N and IL-1β protein expression. *p<0.05.

## Data Availability

All data included in this study are available upon request by contact with the first author or corresponding author.
