# Correction: Identification of ANXA3 as a biomarker associated with pyroptosis in ischemic stroke

**DOI:** 10.1186/s40001-024-01693-y

**Published:** 2024-02-07

**Authors:** Linquan Liu, Yahong Cai, Changqing Deng

**Affiliations:** 1 Chronic Disease Management Department, The First Hospital of Hunan, University of Chinese Medicine, Changsha, 410007 Hunan China; 2 The Key Laboratory of Hunan Province for Integrated Traditional Chinese, and Western Medicine on Prevention and Treatment of Cardio‑Cerebral Diseases, College of Integrated Traditional Chinese and Western Medicine, Hunan University of Chinese Medicine, Changsha, 410208 Hunan China

**Correction: European Journal of Medical Research 2023, 28:596** 10.1186/s40001-023-01564-y

The wrong Supplementary file 6 was originally published with this article [1] as one of the bands from the Western blot in Figure S3 was duplicated from one in Fig. 7; it has now been replaced with the correct file. The original article has been corrected (Additional file 1).

### Supplementary Information

**Additional file 1:** **Figure S3.** OGD/R+LPS promotes cellular pyroptosis. **A** CCK-8; **B** GSDMD-N and IL-1β protein expression. *p <0.05.

## References

[1] Liu L, Cai Y, Deng C (2023). Identification of ANXA3 as a biomarker associated with pyroptosis in ischemic stroke. Eur J Med Res.

