# Supplementary material for: Identification of ANXA3 as a biomarker associated with pyroptosis in ischemic stroke
Source: Eur J Med Res. 2023 Dec 15;28:596. doi: 10.1186/s40001-023-01564-y (PMC10725036; doi:10.1186/s40001-023-01564-y)
Supplement: Supplementary file 1 — Additional file 1: Table S1. Basic information of gene expression profiling. [file 40001_2023_1564_MOESM1_ESM.docx]

| **Table S1. Basic information of gene expression profiling** | | | | | | | |
| --- | --- | --- | --- | --- | --- | --- | --- |
| **Platform** | **GEO Accession ID** | **Samples (Total number)** | **Number of cases** | **Number of controls** | **Country** | **Year** | **Author** |
| GPL570 | GSE58294 | 92 | 69 | 23 | USA | 2014 | Boryana Stamova |
|  | GSE22255 | 40 | 20 | 20 | Portuga | 2011 | Sofa A Oliveira |
|  | GSE66724 | 16 | 8 | 8 | Spain | 2016 | Jose Hermida |
| GPL6883 | GSE16561 | 63 | 39 | 24 | USA | 2010 | Taura L Barr |
|  | GSE37587 | 68 | 34 | 34 | USA | 2015 | Taura L. Barr |
