# Supplementary material for: Identification of ANXA3 as a biomarker associated with pyroptosis in ischemic stroke
Source: Eur J Med Res. 2023 Dec 15;28:596. doi: 10.1186/s40001-023-01564-y (PMC10725036; doi:10.1186/s40001-023-01564-y)
Supplement: Supplementary file 2 — Additional file 2: Table S2. Pyroptosis-related gene sets. [file 40001_2023_1564_MOESM2_ESM.docx]

| **Table S2. Pyroptosis-related gene sets** | |
| --- | --- |
| **Name** | **Name** |
| DHX9 | NAIP |
| AIM2 | NFKB1 |
| APIP | NFKB2 |
| CASP1 | NLRC3 |
| CASP3 | NLRC4 |
| CASP4 | NLRP1 |
| CASP5 | NLRP12 |
| CASP6 | NLRP2 |
| CASP8 | NLRP3 |
| CASP9 | NLRP6 |
| DDX58 | NLRP7 |
| ELANE | NLRP9 |
| GSDMA | NOD1 |
| GSDMB | NOD2 |
| GSDMC | PJVK |
| GSDMD | PLCG1 |
| GSDME | PRKACA |
| IFI16 | SCAF11 |
| IL-18 | TIRAP |
| IL-1β | TNF |
| MAPK8 | GPX4 |
| MAPK9 | IL-6 |
