# Supplementary material for: Identification of ANXA3 as a biomarker associated with pyroptosis in ischemic stroke
Source: Eur J Med Res. 2023 Dec 15;28:596. doi: 10.1186/s40001-023-01564-y (PMC10725036; doi:10.1186/s40001-023-01564-y)
Supplement: Supplementary file 3 — Additional file 3: Table S3. Antibody information. [file 40001_2023_1564_MOESM3_ESM.docx]

| **Table S3. Antibody information** | | | | | |
| --- | --- | --- | --- | --- | --- |
| **Name** | **Product code** | **Dilution Rate** | **Molecular weight** | **Manufacturer** | **Country** |
| ANXA3 | 11804-1-AP | 1:3000 | 36KDa | Proteintech | USA |
| ADM | ab190819 | 1:500 | 20KDa | Abcam | UK |
| ANKRD22 | bs-9748R | 1:1000 | 22KDa | Bioss | China |
| NLRP3 | ab263899 | 1:1000 | 118KDa | Abcam | UK |
| NLRC4 | ab201792 | 1:1000 | 117KDa | Abcam | UK |
| AIM2 | 20590-1-AP | 1:3000 | 39-45KDa | Proteintech | USA |
| GSDMD | MA5-44666 | 1:1000 | 50,30KDa | Thermofisher | USA |
| caspase8 | ab227430 | 1:1000 | 55KDa | Abcam | UK |
| caspase1 | ab179515 | 1:1000 | 45,42,35,12,10KDa | Abcam | UK |
| IL-1β | ab254360 | 1:1000 | 30KDa | Abcam | UK |
| IL-18 | ab191860 | 0.5 μg/ml | 22KDa | Abcam | UK |
| β-actin | 66009-1-Ig | 1:5000 | 42KDa | Proteintech | USA |
| HRP goat anti-mouse IgG | SA00001-1 | 1:5000 | - | Proteintech | USA |
| HRP goat anti-rabbit IgG | SA00001-2 | 1:5000 | - | Proteintech | USA |
