# Supplementary figures and images for: Identification of ANXA3 as a biomarker associated with pyroptosis in ischemic stroke
Source: Eur J Med Res. 2023 Dec 15;28:596. doi: 10.1186/s40001-023-01564-y (PMC10725036; doi:10.1186/s40001-023-01564-y)

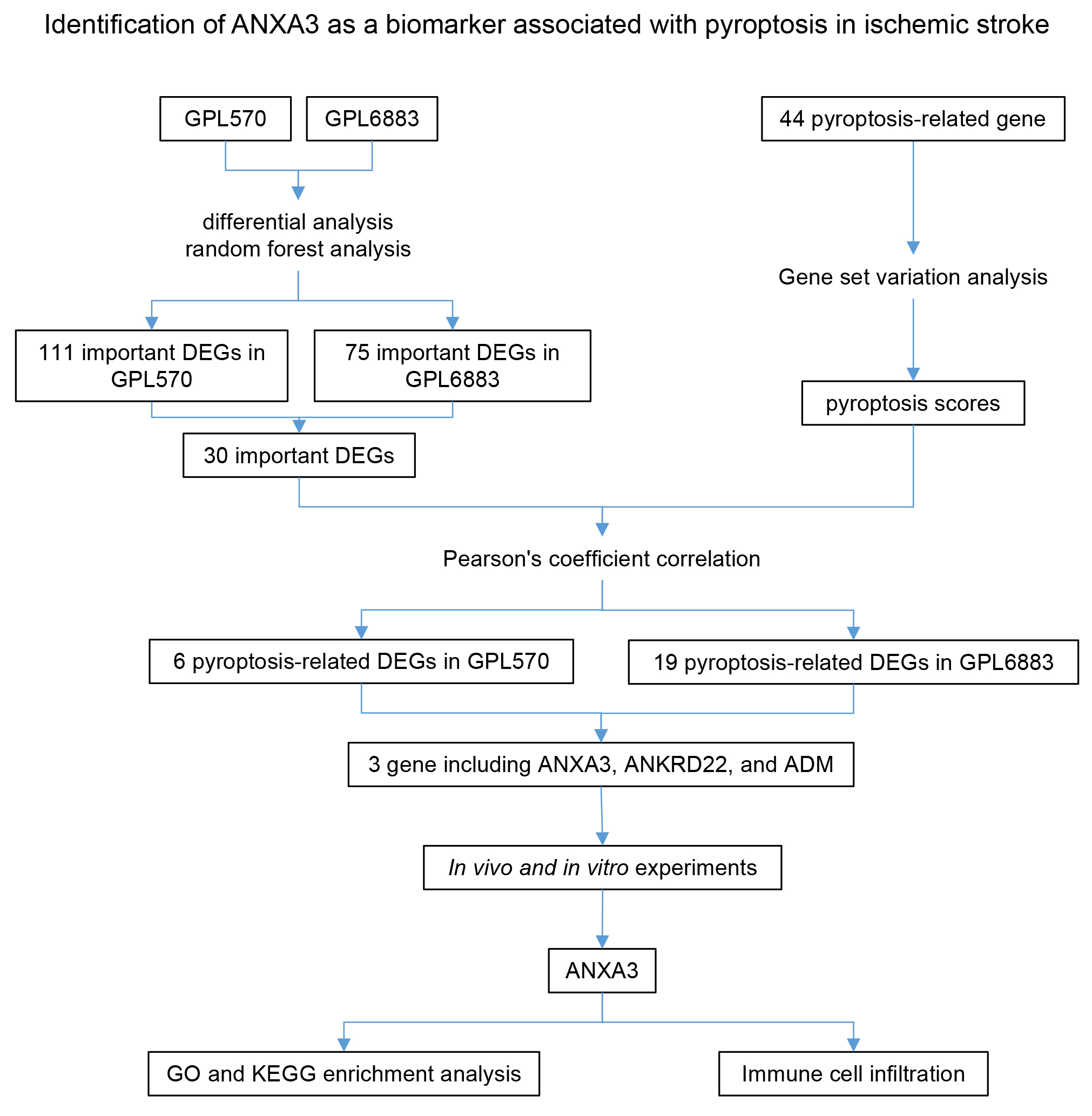

Supplement: Supplementary file 4 — Additional file 4: Fig. S1. Flow diagram of the study. [file 40001_2023_1564_MOESM4_ESM.jpg]

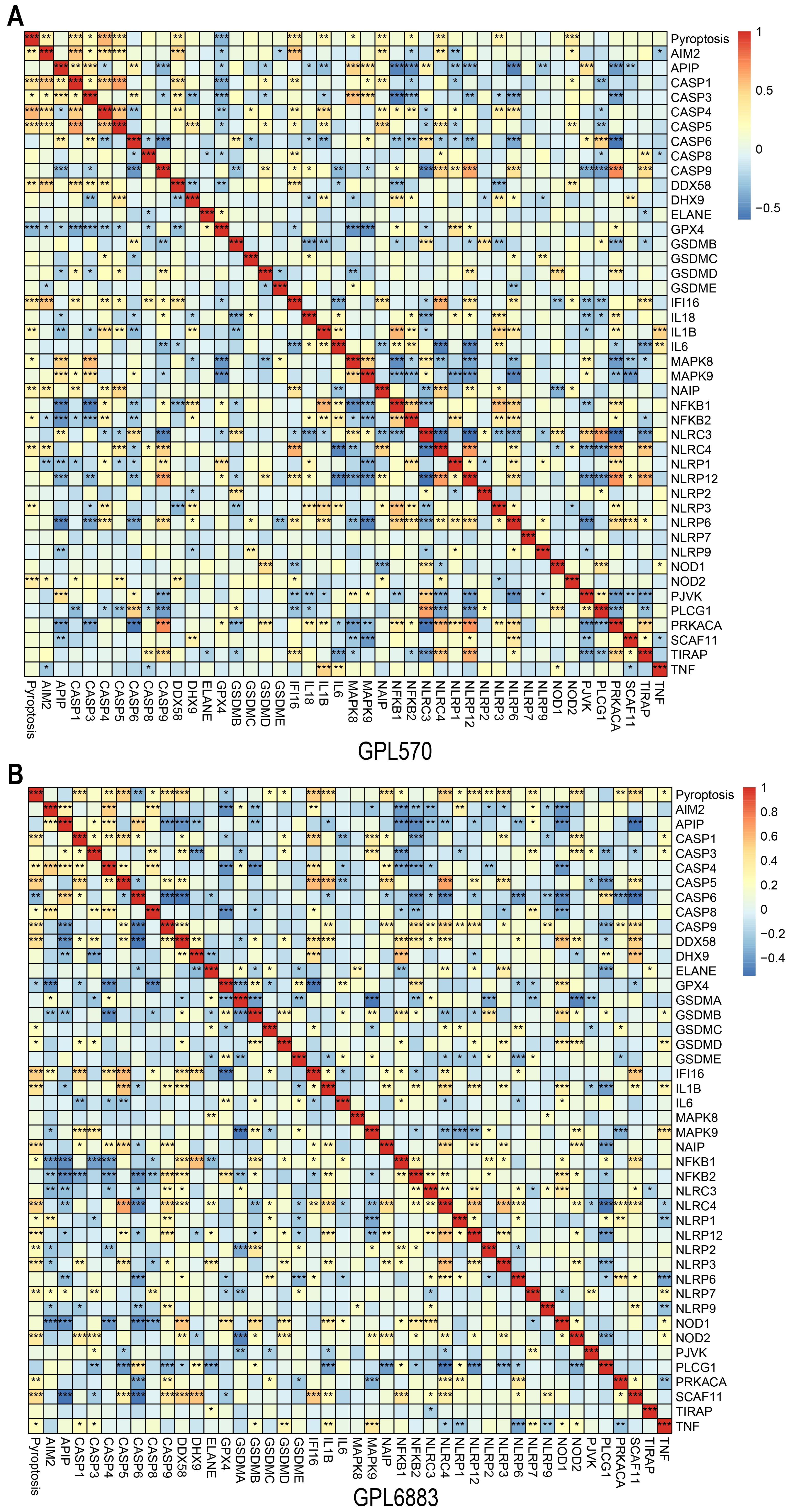

Supplement: Supplementary file 5 — Additional file 5: Fig. S2. Expression correlation analysis of the pyroptosis-related gene set in the GPL570 and GPL6883 datasets. (A) GPL570; (B) GPL6883. [file 40001_2023_1564_MOESM5_ESM.jpg]

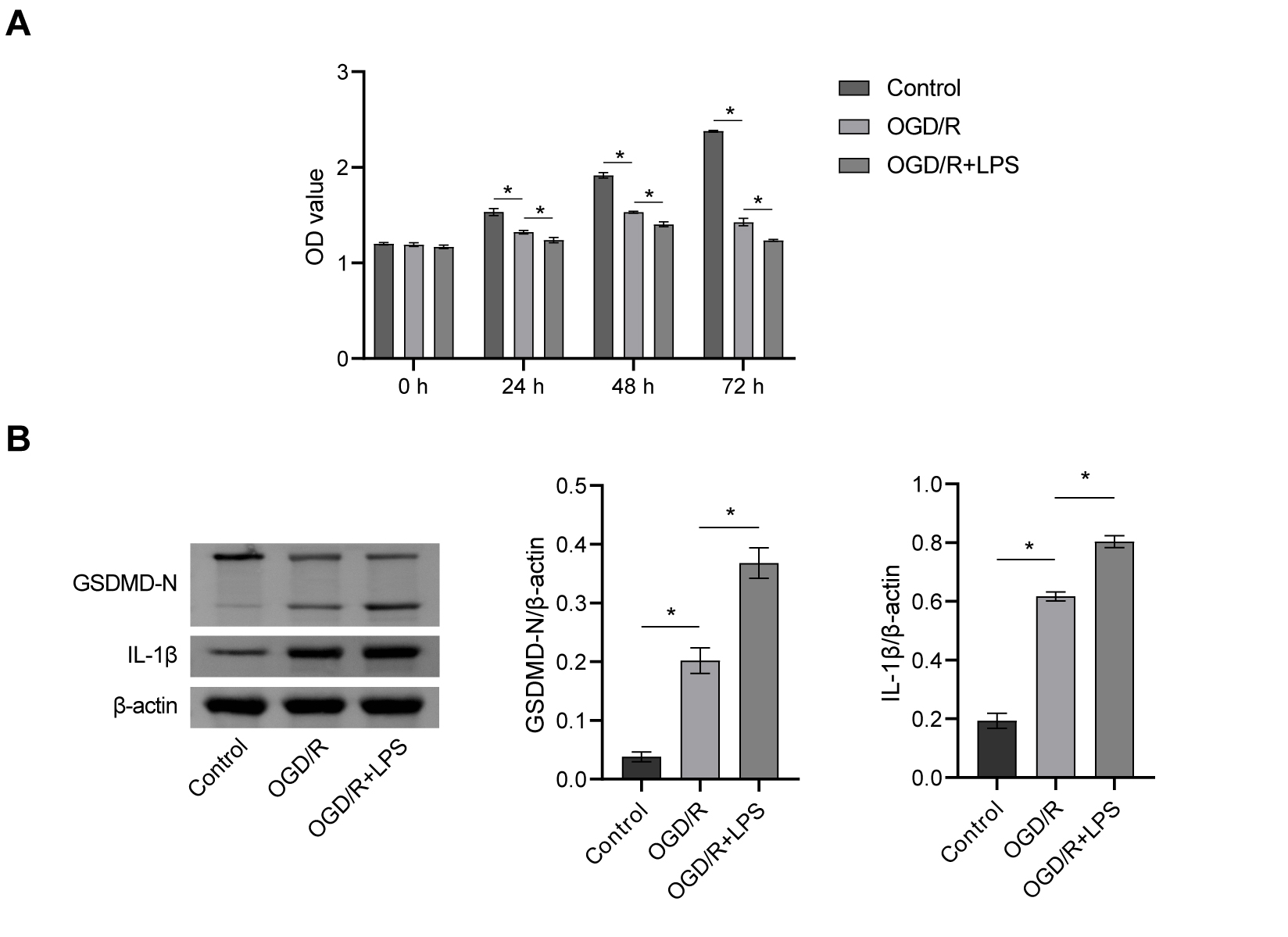

Supplement: Supplementary file 6 — Additional file 6: Fig. S3. OGD/R+LPS promotes cellular pyroptosis. (A) CCK-8; (B) GSDMD-N and IL-1β protein expression. *p<0.05. [file 40001_2023_1564_MOESM6_ESM.jpg]
