# Supplementary figures and images for: Correction: Identification of ANXA3 as a biomarker associated with pyroptosis in ischemic stroke
Source: Eur J Med Res. 2024 Feb 7;29:105. doi: 10.1186/s40001-024-01693-y (PMC10848556; doi:10.1186/s40001-024-01693-y)

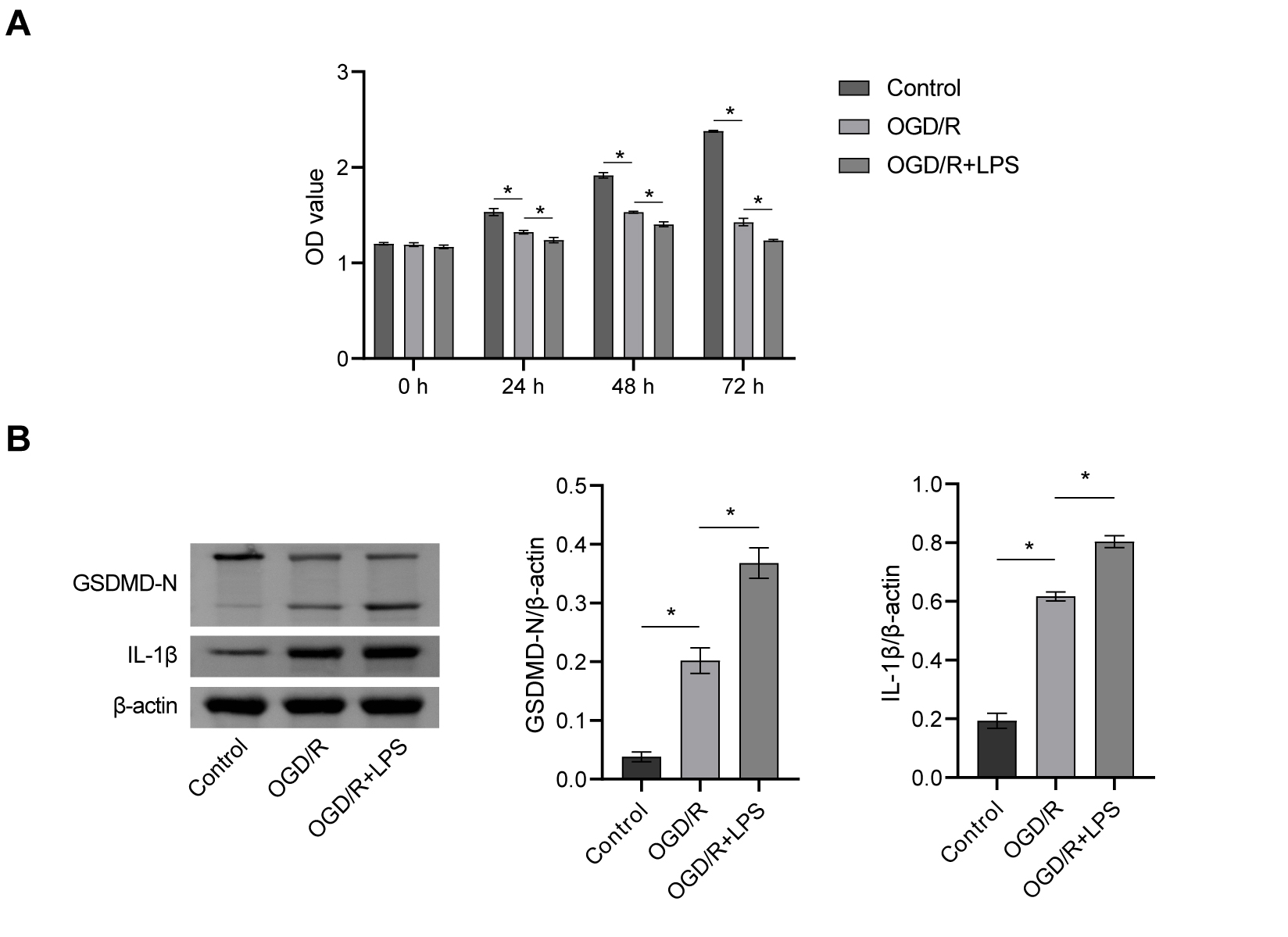

Supplement: Supplementary file 1 — Additional file 1: Figure S3. OGD/R+LPS promotes cellular pyroptosis. A CCK-8; B GSDMD-N and IL-1β protein expression. *p <0.05. [file 40001_2024_1693_MOESM1_ESM.jpg]
